# How I experienced tele-intervention: Qualitative insights from persons who stutter

**DOI:** 10.4102/sajcd.v72i1.1068

**Published:** 2025-01-31

**Authors:** Raadhiyah Hoosain, Shabnam Abdoola, Esedra Krüger, Bhavani Pillay

**Affiliations:** 1 Department of Speech-Language Pathology and Audiology, Faculty of Humanities, University of Pretoria, Pretoria, South Africa; 2 Department of Speech-Language Pathology, United Arab Emirates University, Al Ain, United Arab Emirates

**Keywords:** dysfluency, persons who stutter, speech therapy, stuttering, tele-intervention, speech-language therapist, perspectives, hybrid intervention

## Abstract

**Background:**

Tele-intervention gained popularity, during the coronavirus disease 2019 (COVID-19) pandemic, prompting healthcare providers to adapt to remote service delivery. Research about stuttering treatment via tele-intervention in South Africa is limited. Speech-language therapists (SLTs) require further insights to deliver a well-supported approach for treatment of stuttering using tele-intervention, despite limitations such as technological disruptions, including loadshedding, that impact service reliability.

**Objectives:**

The study aims to explore clients’ experiences with tele-intervention for stuttering therapy, and to provide recommendations to improve service delivery.

**Method:**

Semi-structured interviews were conducted with 11 persons who stutter (PWS) recruited through purposive sampling. Written informed consent was obtained from all participants with experience in both tele-intervention and in-person treatment. Inductive thematic analysis supplemented by descriptive statistics was used to identify patterns and trends.

**Results:**

Four main themes emerged: (1) User experiences and factors shaping perceptions of tele-intervention; (2) technical infrastructure: barriers and facilitators; (3) financial and access considerations and (4) in-person treatment experience compared to tele-intervention user experience. Likert scale ratings indicated no considerable difference in preferences between tele-intervention and in-person treatment.

**Conclusion:**

Participants’ diverse experiences highlighted tele-intervention’s benefits and challenges for stuttering therapy. While limitations exist, findings inform service enhancement in South Africa, emphasising the importance of users’ perspectives in tele-intervention design.

**Contribution:**

Insights from PWS can be used in informing clinical practice, aiding SLTs in meeting the needs of PWS and guiding best practice. Tele-intervention should be integrated into a hybrid intervention model that PWS prefer.

## Introduction

Tele-intervention, the use of telecommunications technology for the delivery of speech-language therapy (SLT) services, has recently gained popularity as a means of linking the clinician to the client at a distance (Weidner & Lowman, 2020). Technology advances and the onset of the COVID-19 pandemic accelerated drastic changes in service delivery where healthcare professionals had to adapt to the unique and unprecedented times by providing services via tele-intervention (Aggarwal et al., 2020; Eslami Jahromi & Ahmadian, 2018). The use of tele-intervention among SLTs in private practice has increased from 10% pre-coronavirus disease 2019 (pre-COVID-19) to 85% in 2020, indicating the potential widespread use of tele-intervention in the field (ASHA, 2020). Within the field of speech-language pathology, stuttering has arguably been a subject of tele-intervention research, in which outcomes of clinical trials indicate that stuttering is well-suited to treatment via tele-intervention (Santayana et al., 2021).

Persons who stutter (PWS) often seek advice and/or the initiation of services from SLTs to reduce negative feelings associated with their dysfluencies, which may have an impact on their quality of life in a variety of contexts (ASHA, 2018). However, with treatment provision inequities as a result of accessibility and availability barriers, PWS often experience challenges seeking treatment (Erickson et al., 2016; McGill et al., 2019). While traditionally, stuttering therapy has been provided in-person, alternative delivery methods such as tele-intervention provide advantages such as promoting the participation of PWS in therapy sessions by utilising videoconferencing and using social networks to overcome barriers (Bayati & Ayatollahi, 2021).

Besides offering a greater outreach of services and increased convenience, studies investigating tele-intervention reported its success as a valid and effective modality for the delivery of stuttering treatment across different age groups (Lam et al., 2021). Clinical research for stuttering treatment programmes such as the Lidcombe programme (Onslow et al., 2020), Camperdown Programme (Onslow et al., 2018) and integrated approaches have established the viability and efficacy of tele-intervention showing a reduction in stuttering severity in pre-schoolers (Bridgman et al., 2016; McGill et al., 2019). Additional studies describe a range of perceived advantages of tele-intervention such as improved scheduling flexibility, time- and travel convenience, and overall satisfaction with the delivery method (Almathami et al., 2020; Chaudhary et al., 2021; McGill & Schroth, 2020; Tar-Mahomed & Kater, 2022). Small-scale studies of treatment outcomes for clients comparing in-person and tele-intervention approaches have found that tele-intervention may be equally as effective as the in-person method of service delivery for adults who stutter (Cangi & Toğram, 2020; Eslami Jahromi & Ahmadian, 2018). Although parents’ experiences have been documented, there are many important questions dealing with the in-depth experiences of PWS within a varied age range and context, which are yet to be explored and addressed.

In contrast, concerns raised about poor tele-intervention engagement, ineffective communication between SLTs and parents and technical difficulties were identified as limitations contributing to reduced client acceptance of the delivery method (Lam et al., 2021; Santayana et al., 2021). Recent findings suggest improving technical infrastructure, training SLTs on tele-intervention implementation and providing the necessary requirements for the implementation of tele-intervention to improve accessibility, increase effectiveness and promote online participation (Bradford et al., 2018; Mohan et al., 2017). However, because of differing health-related policies, technical infrastructure, culture and obstacles varying across countries, client experiences about using tele-intervention may also differ based on a given context (Tar-Mahomed & Kater, 2022). For example, according to South Africa Statista (2021), only 61% of South Africans have internet access in their homes and disparities in access to services and technology coupled with high data costs, unreliable connectivity and interrupted electricity supplies, may influence access to tele-intervention services (Watermeyer et al., 2022). Further contributing to challenges experienced by PWS seeking treatment, capacity versus demand for SLT delivery continues to be inequitable for the South African population, contributing to accessibility barriers (Abrahams et al., 2019). This may differ from high-income countries with appropriate technological infrastructure, compared to upper-middle income countries such as South Africa’s infrastructure (Govender et al., 2022; Khoza-Shangase et al., 2021).

Studies on tele-intervention highlight an SLT- and parent-centric perspective, with limited data and pertinent questions about SLT online services from client perspectives within a varied age range remaining unanswered (Campbell & Goldstein, 2022). Furthermore, existing research on client experiences, shows that clients have mixed experiences with a tele-intervention service delivery method (Eslami Jahromi et al., 2022). Clients’ concerns must be addressed as the wider acceptability and implementation of tele-intervention depend on improving the service delivery method (Bayati & Ayatollahi, 2021). Concerns-based client experiences may best be addressed using qualitative methods as qualitative studies may provide insight into ethically informing clinical practice (Karani & Mupawose, 2020). This study could provide an enhanced understanding of clients’ experiences concerning tele-intervention and contribute to the improved implementation of a service delivery model for stuttering.

## Research methods and design

### Design

A mixed-methods approach was employed utilising a semi-structured interview and questions including Likert scale ratings. The research design was descriptive and phenomenological in nature, as the study aimed to describe client experiences of tele-intervention. In addition, Likert scale ratings were utilised to quantify PWS’ experiences with tele-intervention for stuttering, enriching the study with quantitative data.

### Setting

The research context involved remote online interviews with PWS receiving tele-intervention services for stuttering from the Speech Therapy and Audiology department at the University of Pretoria. Interviews were conducted on the online platform Microsoft Teams and each participant engaged in a single interview session facilitated by the first author.

### Population and sampling strategy

A purposive sampling method was used to select PWS who have received tele-intervention services. Permission was obtained to recruit members from the Clinic of Fluency Disorders from the Department of Speech-Language Pathology and Audiology at the University of Pretoria. An information letter was provided to prospective participants and informed consent was obtained. A total of 11 participants were included based on recruitment capacity and specific inclusion criteria.

The following inclusion criteria were applied: (1) PWS aged 12 years and older, (2) PWS who have received a minimum of four treatment sessions via tele-intervention, (3) PWS with fluency disorders ranging from mild (below 5% of syllables stuttered) to severe (above 20% of syllables stuttered) and (4) PWS with access to devices on which they can access the Internet. Children aged 12 years and older were included in this study as they have the cognitive ability to comprehend abstract questions and, within this age group, stuttering and associated patterns are well-established and can be effectively addressed through therapy (Guitar, 2018). Table 1 presents detailed information about the 11 participants recruited.

**TABLE 1:** Participants’ demographics.

Participant no.	Age (years)	Age category	Age range (years)	Gender	Highest level of education	Total duration of telepractice services received	Frequency of telepractice treatment sessions	Duration of telepractice treatment sessions (minutes)
P1	22	Young Adult	19–30	Male	Undergraduate qualification	More than a year	Once a week	60
P2	16	Adolescent	13–18	Male	High school	More than a year	More than once a week	30
P3	18	Adolescent	13–18	Male	High school	More than a year	Once a week	60
P4	28	Young Adult	19–30	Male	Undergraduate qualification	More than a year	Once a week	60
P5	45	Adult	31–59	Male	Postgraduate qualification	More than a year	Bimonthly	30–45
P6	26	Young Adult	19–30	Male	Postgraduate qualification	More than a year	Bimonthly	> 60
P7	16	Adolescent	13–18	Male	High school	More than a year	Once a week	30
P8	35	Adult	31–59	Male	Primary school	More than a year	Once a week	60
P9	17	Adolescent	13–18	Male	High school	More than a year	Once a week	60
P10	32	Adult	31–59	Male	Postgraduate qualification	More than a year	Once a week	60
P11	56	Adult	31–59	Female	High school	Less than a year	Once a week	30–45

### Data collection

Participants were interviewed via Microsoft Teams using a semi-structured interview for approximately 40 min. The interview schedule was compiled, extrapolated and adapted from interviews and Likert responses based on studies conducted by Eslami Jahromi et al. (2020) and McGill et al. (2019). The researcher adapted relevant questions and responses that align with the aim of this study. The adapted interview schedule was then utilised in a pilot study to identify any adaptations required. This involved simplifying the language used in the questions, removing technical terminology and formulating additional questions to gain further insight into PWS’ experiences regarding tele-intervention for stuttering. The interview schedule contained five sections, each focusing on different aspects relating to client experiences of tele-intervention. The five sections included: (1) demographic information; (2) tele-intervention implementation; (3) technical aspects relating to tele-intervention; (4) client satisfaction with tele-intervention and (5) comparison of tele-intervention to face-to-face treatment. Likert scale questions were included in sections three, four and five to obtain quantitative data where participants were expected to rate their experiences with tele-intervention using a 5-point Likert scale. (e.g. 1 = strongly disagree, 5 = strongly agree).

### Data analysis

Qualitative data were analysed following Braun and Clarke’s (2014) thematic analysis guidelines. The researcher recorded and transcribed the interviews, which were reviewed to enhance data familiarity. ATLAS.ti software facilitated initial line-by-line semantic coding of participant transcriptions, as well as the reviewing, listing, categorising, comparing and management of codes. Subsequently, semantic codes were refined to generate latent codes that were classified into themes organised around a primary concept or idea shared by the participants. To ensure dependability, co-authors reviewed themes following Braun and Clarke’s (2014) recommendations. All authors contributed to refining theme definitions and names. Descriptive statistics summarised responses to direct (yes or no) questions and frequency distributions were used to gain insight into Likert scale responses.

### Ethical considerations

Institutional ethical clearance was obtained from the University of Pretoria Faculty of Humanities Research Ethics Committee (HUM023/0622). All participants gave written informed consent prior to the commencement of interviews. In the case of adolescents aged between 12 years and 17 years old, assent forms were completed, and parents or legal guardians provided written consent and were present during the interview.

## Results

**FIGURE 1:**
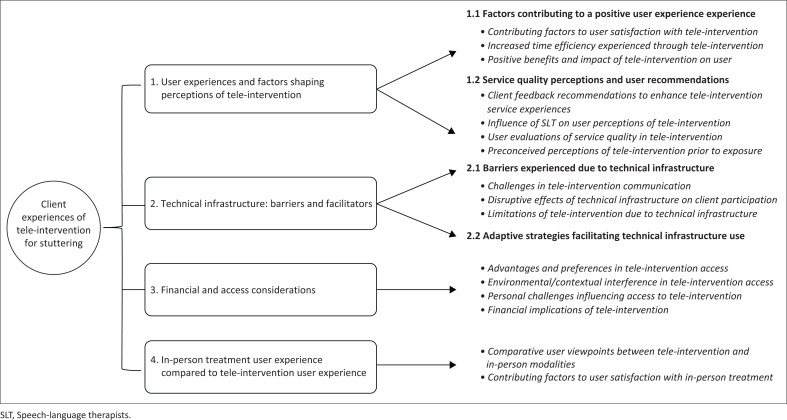
Four major themes and subthemes.

Four main themes emerged from participant interviews: (1) tele-intervention user experiences and factors shaping perceptions (2) technical infrastructure: barriers and facilitators (3) financial and access considerations and (4) in-person treatment experience compared to tele-intervention user experience (Figure 1). In addition to their interview responses, participants rated their experiences with tele-intervention using a 5-point Likert scale. Responses from quantitative data have been included in the thematic descriptions, which follow next.

### Theme 1: User experiences and factors shaping perceptions of tele-intervention

#### Subtheme 1.1: Factors contributing to a positive user experience

Ten participants (91%) reported benefits of tele-intervention in terms of scheduling flexibility, integration into daily routines and reduced travel time. Specifically, scheduling convenience emerged as a primary advantage, highlighted by eight participants (73%), who appreciated the ease of flexibility to plan sessions around personal schedules. One of the participants commented:

‘It was pretty easy to fit those meetings [*tele-intervention*] into my time schedule and day around classes since I was always nearby a device capable of joining those meetings.’ (Participant 1)

In addition, six participants (55%) noticed the time-saving aspect of tele-intervention, with reduced travel time:

‘… you don’t have to waste time travelling back and forth, so you can save that time to do other stuff for yourself … those two hours of tele-intervention, if it was going to be done in-person, maybe it was going to be expanded into three or four hours.’ (Participant 6)

Four participants (36%) identified that receiving tele-intervention services in a familiar setting, such as in the comfort of their own home, contributed to an overall positive experience of the medium:

‘I think it’s a more relaxed environment to focus on therapy and you can set it up so that you are not interrupted by anything so that works really well.’ (Participant 5)

Ten participants (91%) reflected on their perceived improvements in fluency as a result of tele-intervention exposure. Their statements were as follows:

‘Well at first I used to stutter a lot and as soon as I started the therapy [*tele-intervention*], as time went on, I actually improved more and more … like drastically improved … so it really helped me a lot.’ (Participant 2)

‘According to me the services are helping because I am able to apply different strategies that the therapists taught me to tackle stuttering.’ (Participant 3)

Ten participants (91%) found an additional positive perception of tele-intervention: the opportunity to become comfortable and proficient in using technology. One participant remarked:

‘My confidence in working with a laptop, it has increased immensely because I was actually forced to use this medium, so it just gave me lots and lots of confidence to work with the laptop.’ (Participant 11)

In addition to these insights, participants rated their experience of comfort with tele-intervention via Likert responses. In terms of comfort with technology before tele-intervention exposure, participants’ ratings ranged from 1 (*very uncomfortable*) to 5 (*very comfortable; M* = 3.7). After exposure to tele-intervention, participants’ comfort with technology ranged from 3 (*neutral*) to 5 (*very comfortable; M* = 4.1). On average, participant responses were neutral (*M* = 3.1) concerning comfort with online platform use before tele-intervention exposure compared to an increase in comfort (*M* = 4.3) with online platforms after exposure to tele-intervention. Nine (82%) participants expressed their satisfaction with tele-intervention with an average Likert rating of 4 (*satisfied; M* = 4.1). Participants 3 and 4 (*n* = 2; 18%) rated their satisfaction with tele-intervention as 2 (unsatisfied) and 3 (neutral), respectively, expressing that they prefer practicing therapy techniques in-person.

#### Subtheme 1.2: Service quality perceptions and user recommendations

All participants (*n* = 11; 100%) positively evaluated the quality of services they received. Speech-language therapists contributed to participant perceptions of service quality as the following influential factors were identified: the relationship with their SLT, how the SLT structured therapy online and the SLT’s ability to use technology. Participants indicated a high level of agreement (*M* = 4.6) regarding maintaining a friendly and positive relationship with their SLT, rating between 4 (*agree*) and 5 (*strongly agree; M* = 4.6). Concerning SLT’s influence on service quality perceptions, one participant stated:

‘It was a tremendous experience for me to do these online sessions but also I think that the therapist’s they are the … how should I say … the foundation of the therapy.’ (Participant 11)

The majority of participants did not agree that their SLT was disengaged during tele-intervention with responses ranging from 1 (*strongly disagree*) to 3 (*neutral; M* = 1.7). For confidence in their SLTs’ ability to navigate the technical components of tele-intervention, ratings ranged from 3 (*neutral*) to 5 (*strongly agree; M* = 4.0). When evaluating their satisfaction with SLT’s use of online platforms to deliver tele-intervention, participant ratings ranged from 2 (*unsatisfied*) to 5 (*very satisfied*; *M* = 3.8).

Initially, six participants (55%) discussed uncertainties about their expectations and perceptions of tele-intervention owing to their unfamiliarity with the service delivery method. However, with increased exposure, all participants overcame their initial hesitations and reported enhanced perceptions of tele-intervention service quality as follows:

‘Well, I was hesitant about online speech therapy before starting. I wasn’t sure if it was going to work or how the sessions are going to go.’ (Participant 1)

‘It was great. The online classes were great actually. I enjoyed every session … at first it was a bit complicated then I got the hang of it.’ (Participant 2)

Perceptions of high quality of service were linked to participants’ confidence that their primary reasons for treatment can be addressed via tele-intervention, with participant ratings ranging from 2 (*disagree*) to 5 (*strongly agree; M* = 4.0). Several participants provided insightful suggestions such as an integration of in-person and tele-intervention methods and engagement with unfamiliar communication partners for the improvement of tele-intervention as seen next:

‘I think for online therapy to improve, therapists should invite other people that are outside [*unfamiliar communication partners*] to engage with them online.’ (Participant 4)

‘If there was anything I could add … the main part missing is the physical face-to-face communication.’ (Participant 6)

Seven participants (64%) felt that there was no need for improvements to be made to their devices or software platforms for an enhanced online experience. On the contrary, three participants (27%) felt strongly that a fast device and a reliable internet connection is required to have a positive experience of tele-intervention.

### Theme 2: Technical infrastructure: Barriers and facilitators

#### Subtheme 2.1: Barriers experienced due to technical infrastructure

Participants reported difficulties with the network connections and unreliable electricity supply. One participant said:

‘Two things are the problem. It’s the network and the electricity cuts [*loadshedding*].’ (Participant 3)

Poor network connections contributed to problems such as lag and poor sound and image quality (*n* = 8; 73%). Six participants (55%) found that poor network connections resulted in disruptive effects on their participation in therapy sessions, reducing the amount of time allocated to therapy as well as experiencing negative feelings such as disappointment, panic and nervousness as stated here:

‘If I did experience those glitches I would get into the meeting a bit more stressed and a bit more nervous and the session would be a bit more challenging.’ (Participant 1)

‘The disruptions make your time be short so that you end up not doing what was planned for the day.’ (Participant 6)

Services were further disrupted by electricity interruptions (loadshedding) experienced by almost half of the participants (*n* = 5; 45%). Five participants (45%) reported the following limitations in communication via a tele-intervention context; delays in communication exchange, a lack of visual input such as making eye contact and a reduced ability to express emotions via tele-intervention:

‘Sometimes I’ll be asking questions that are practical but then because it’s online, it’s hard for them [*the therapist*] to answer in a practical or demonstrative way.’ (Participant 4)

‘There are some things that you don’t focus on as much in therapy based on the setup, one of the things is making eye contact.’ (Participant 5)

‘Online for me, I’m not free, I’m not open. I cannot express my emotions and stuff online.’ (Participant 10)

#### Subtheme 2.2: Adaptive strategies facilitating technical infrastructure use

On the contrary, despite reporting on difficulties with unreliable electricity supply, network issues and poor sound and video input, the majority of participants (*n* = 9; 82%) indicated that these challenges did not impact their active participation during their online therapy sessions. Two participants commented:

‘I would say most times it [*the sound*] was clear I was easily able to understand them at all times.’ (Participant 1)

‘After like a minute or so [*of network problems*] it would be okay and then we would continue the session.’ (Participant 2)

Participants described overcoming these disruptions by briefly waiting for network improvements, reducing bandwidth usage by deactivating video input on online platforms or by accessing tele-intervention with an alternate device as follows:

‘Sometimes when I could not see her then we just worked without the video and had a productive session.’ (Participant 5)

‘… but even if they [*the SLTs*] used their cell phone to communicate with us during loadshedding [*electricity shortages*] you almost didn’t notice the difference.’ (Participant 11)

Eight participants (73%) credited their ability to overcome network issues by relying on better sound than video quality, identifying sound input as the main priority for active participation during therapy sessions. One participant commented:

‘The sound quality was quite good and because of that I didn’t have any problem without the video and I was able to participate actively in those sessions.’ (Participant 6)

### Theme 3: Financial and access considerations

Participants provided insights regarding the accessibility of tele-intervention, including the types of devices used and the financial implications associated with accessing the service delivery method. All participants used laptops and some (*n* = 5; 45%) used a laptop and mobile phone. Mobile phones were utilised during electricity outages and when participants needed a device readily available to facilitate multitasking in different environments. Three participants (27%) described the distracting effects of a mobile device and how using outdated, slow devices that required regular software updates hindered their participation in therapy. One participant commented:

‘I think on cell phones, trying to avoid phone calls while in the meeting is distracting.’ (Participant 4)

On the contrary, most participants (*n* = 8; 73%) indicated that no device improvements to a laptop were necessary to enhance their tele-intervention experience:

‘… the computer has all the tools available to receive those [*therapy*] materials and host the meetings and I’m also able to print materials out if it’s needed with the computer’s capabilities so I’d say it was an appropriate platform.’ (Participant 1)

Overall, all participants regarded devices positively, emphasising that these devices were essential for access to tele-intervention as described next:

‘I think devices have done a lot in terms of accessibility for speech therapy … that time it was not easy for me to access [*intervention*] in-person and it [*tele-intervention*] made it [*speech therapy*] accessible to me.’ (Participant 6)

Participants (*n* = 8; 73%) highlighted the advantage of decreased costs because of reduced travel costs. Participants explained:

‘Face-to-face was more expensive because of petrol, and we also saved time doing therapy online. The laptop helped to overcome the distance barrier so that I could have therapy.’ (Participant 7)

‘If we didn’t have a computer and we didn’t have Teams we would have probably travelled once a week … and now with petrol costs it would have been highly expensive, but with this [*tele-intervention*], financially it’s unbelievable and makes so much sense.’ (Participant 11)

On the contrary, three participants (27%) mentioned mobile data usage resulted in increased costs. One participant stated:

‘I feel I was spending more money because I was the one who was doing everything on my own … loading my own data and it [*data costs*] was high at that time. There are no specials for data.’ (Participant 10)

### Theme 4: In-person treatment user experience compared to tele-intervention user experience

All participants interviewed had received therapy in-person and via tele-intervention and therefore provided insights regarding the two service delivery methods. Regarding service delivery preferences, five participants (46%) expressed their preference for in-person therapy. Three participants (27%) stated their preference for tele-intervention and the remainder of participants (*n* = 3; 27%) had no specific preference between the two service delivery models. When providing comparisons between the two modalities, participants found specific factors, which contributed to a positive perspective of in-person treatment. These factors included: the ability to participate in varied contexts, receiving increased practical exposure to treatment techniques, a lack of technical disruptions and reduced exposure to distractions such as background noise. Describing the additional variety of exercises available and ease of communication with in-person therapy, participants expressed:

‘There’s a lot of extra variety and different exercises you can do in-person that you can’t really do online and it’s sometimes easier to discuss techniques and apply those techniques in practice in person.’ (Participant 1)

‘I think the in-person sessions were quite good and helpful because some things you can do online, but certain things require you to be there in person to be able to do them well or to take you out of your comfort place and put you in a real place.’ (Participant 6)

Participant 3 highlighted the contrast in interruptions and distractions between modalities, stating:

‘And with face-to-face, I was improving much better, but with Zoom, the networks and the noises outside were disruptive … with face-to-face we [*the client and the SLT*] can understand each other very well.’

Similarly, Participant 4 emphasised the absence of technical disruptions during face-to-face sessions, saying, ‘with face-to-face, there were no worries of technical problems and there were no experiences of disruptions’.

Despite their inclination towards in-person treatment, all participants expressed that they would still recommend tele-intervention to others who stutter. In terms of reaching goals through a specific service delivery model, participants reported an indifference between in-person delivery (*M* = 3.5) and tele-intervention (*M* = 3.6). When comparing perceived progress in fluency achieved through two different modalities, a participant reflected:

‘My speech was much worse before starting online therapy but I made progress during online therapy so I think I can make as much progress with tele-intervention as an in-person delivery model.’ (Participant 1)

## Discussion

Persons who stutter voiced perspectives on their experiences of tele-intervention for stuttering in South Africa. They highlighted challenges related to technical infrastructure and personal challenges. However, despite obstacles, positive experiences were emphasised, making tele-intervention a viable option to pursue in South Africa.

### User experiences and factors shaping perceptions of tele-intervention

Participants expressed satisfaction with tele-intervention, echoing previous research indicating factors such as accessibility, faster access to treatment and cost and time savings, ultimately contribute to increased client participation, particularly among participants with limited access to healthcare and resources (Eslami Jahromi et al., 2022; Govender et al., 2022; Piron, 2017). Accessing therapy within a familiar setting such as the home environment reduced client anxiety. A positive therapy environment may foster improved fluency and enhance self-image for PWS (Briley et al., 2023; McGill & Schroth, 2020). Speech-language therapists should advocate for therapy contexts, which promote confidence and positive attitudes toward communication for PWS.

Increased proficiency with technology through consistent tele-intervention exposure facilitated participants’ acceptance and adaptation to the medium. Consistent with previous research conducted by Chaudhary et al., (2021) and McGill et al. (2019), participants ultimately adapted to the medium despite initial hesitations about tele-intervention efficacy, demonstrating its adaptability and effectiveness in meeting their primary treatment goals. Improved technology proficiency not only enriches therapy experiences but also reinforces client confidence in technology use (Almathami et al., 2020; Fairweather et al., 2016). Notably, perceptions of enhanced SLT technology proficiency are strongly associated with high service quality ratings, instilling confidence in clients and fostering increased therapeutic engagement and positive client-therapist rapport (Békés et al., 2023). Speech-language therapists should acquire tele-intervention skills to foster effective engagement and interaction between therapist and client via an online medium.

In a similar study conducted by Almathami et al. (2020), participants’ recommendations highlight the impact of a stable internet connection on clients’ perceptions of tele-intervention. Additionally, others recommended a hybrid approach, which combines in-person and tele-intervention methods to provide increased practical exposure. A study by Govender et al. (2022) shows that a combination of in-person and tele-intervention methods can increase access to quality patient-centred care. These insights are vital for SLTs to consider when designing interventions that foster positive experiences, reduce anxiety and ultimately enhance fluency outcomes in tele-intervention.

### Technical infrastructure: Barriers and facilitators

This study identified technological disruptions as the primary barrier to effective delivery of tele-intervention, similar to a study conducted by Annis et al. (2020). The importance of high internet speed for client acceptance and satisfaction in tele-intervention is underscored by participants’ negative remarks and Likert ratings about network connections (Tar-Mahomed & Kater, 2022). This further aligns with findings on network reliability and its direct impact on client satisfaction (Chaudhary et al., 2021). Furthermore, participants reported limitations in communication, including delays in communication exchange, lack of practical exposure to treatment techniques, a reduced ability to express emotions and difficulties in replicating non-verbal communication such as making eye contact. Non-verbal communication including visual features is needed to build rapport between client and therapist (Lam et al., 2021). Difficulties and experiencing disruptions in efficient communication caused negative feelings among participants ultimately resulting in a preference for face-to-face therapy, similarly illustrated in previous research (Eslami Jahromi et al., 2022). These barriers highlight the potential obstacle that South Africa’s technical infrastructure may pose to the widespread implementation and acceptance of tele-intervention, especially in poorly resourced communities (Govender et al., 2022). Speech-language therapists should consider the feasibility and efficacy of tele-intervention for each client, considering individual contextual factors such as technological access and its implications on clients.

Despite challenges, the majority of participants found ways to actively participate in therapy. Similar to research conducted by Wootton et al. (2020), this study found that turning off video input and prioritising sound quality to reduce bandwidth usage is an effective adaptive strategy to overcome connectivity issues. Participants’ flexibility and resilience in overcoming technical difficulties highlight the importance of participants’ commitment and ability to provide practical solutions to enhance tele-intervention experiences (Wootton et al., 2020). Speech-language therapists should ensure that they are informed on practical solutions that can be implemented to overcome technical difficulties. By implementing these strategies, tele-intervention may be a viable option in upper-middle income countries such as South Africa, despite unreliable technical infrastructure.

### Financial and access considerations

Access to devices and data is essential in enabling clients to receive tele-intervention. In line with previous research, participants who could use alternative devices, like mobile phones, found it easier to access tele-intervention sessions, especially during electricity shortages (Wootton et al., 2020). This demonstrates the value of device flexibility, with mobile phones demonstrating their ability to fulfil computer-like functions, further enabling easy access to intervention via mobile devices. Similar to research conducted by Chaudhary et al. (2021), this study highlights the fact that implementation of tele-intervention services via mobile devices provides a cost-effective solution to reducing travel costs and receiving therapy instantaneously. Speech-language therapists play a crucial role in advocating for increased access to tele-intervention by emphasising the significance of device flexibility. While easy accessibility and reduced costs via Wi-Fi was reported, increased costs were associated with mobile data usage, which highlights the disparities among clients in terms of access to the Internet. In a similar study, access to data was influenced by participants’ socio-economic status (Tar-Mahomed & Kater, 2022). When implementing adaptive strategies, SLTs should prioritise accessibility, financial considerations and participant satisfaction to ensure equitable service provisions and engagement.

### In-person treatment user experience compared to tele-intervention user experience

Participants highlighted benefits of in-person therapy, emphasising the physical presence and interaction inherent in face-to-face sessions provide unique benefits that are not fully replicated in tele-intervention settings (Almathami et al., 2020; Eslami Jahromi et al., 2022). In addition, participants commented on the absence of technical disruptions and distractions in face-to-face therapy contributed to a focused and conducive learning environment. Despite their inclination towards in-person treatment, all participants expressed a willingness to recommend tele-intervention to others who stutter, indicating recognition of its potential benefits. Moreover, participants reported similar progress in fluency through both modalities, highlighting the effectiveness of tele-intervention in facilitating improvements in speech fluency as seen in previous research (Chaudhary et al., 2021).

While in-person therapy may offer certain advantages, such as increased practical exposure and reduced technical disruptions, tele-intervention remains a viable and potentially effective alternative or supplement to in-person therapy (Eslami Jahromi et al., 2022; McGill & Schroth, 2020; Tar-Mahomed & Kater, 2022). A combination of in-person therapy and tele-intervention could be effective for improving speech fluency. This hybrid approach that participants recommended, may maximise engagement and effectiveness.

### Strengths and limitations

While the study provides valuable insights into PWS experiences with tele-intervention for stuttering, certain limitations must be acknowledged, including the reliance on self-report data, potentially introducing bias. The small sample in this study may limit the generalisability of findings. The focus on client experiences from an individual clinic may not fully capture the breadth of perspectives across diverse regions and cultural contexts. Future research endeavours should include larger and more diverse samples, employing mixed-methods approaches to gain a comprehensive understanding of tele-intervention experiences among individuals who stutter. Continuous research efforts and ongoing evaluation are indispensable in refining tele-intervention approaches and ensuring their sustained effectiveness and relevance in stuttering intervention.

## Conclusion

This study integrates experiences and voices of PWS with previous research to provide evidence-based recommendations for SLTs delivering tele-intervention services for stuttering, emphasising the crucial role of user input in service design and implementation. Persons who stutter experiences in this study have highlighted important clinical considerations for the management of stuttering via tele-intervention in upper-middle income countries such as South Africa, informing recommendations for SLTs to promote tele-intervention and to consider client preferences regarding hybrid approaches during stuttering intervention.
